# Shared Psychotic Disorder in Two Adolescent Sisters: A Case Report

**DOI:** 10.1192/j.eurpsy.2026.11169

**Published:** 2026-07-31

**Authors:** I. Bouamoud, Z. El Maataoui, H. Kisra

**Affiliations:** Pedopsychiatry, Arrazi Hospital, Salé, Morocco

## Abstract

**Introduction:**

Shared psychotic disorder (*folie à deux*) is a rare psychiatric condition in which a delusional belief is transmitted from a psychotic individual (the inducer) to another person (the induced), typically within a close and isolated relationship. Though mostly described in adult pairs, pediatric and adolescent cases remain underreported and diagnostically challenging.

**Objectives:**

This case report aims to describe the clinical presentation and diagnostic challenges of a shared psychotic disorder in two adolescent sisters, and to review the relevant literature regarding its classification, risk factors, and management.

**Methods:**

We conducted a detailed clinical observation of two sisters, aged 16 and 13, admitted for behavioral disturbances and persecutory delusions. Psychiatric evaluations were carried out, differential diagnoses were explored, and a review of the literature on shared psychotic disorder was undertaken to contextualize the case.

**Results:**

The elder sister, Aya, exhibited a mystic-religious persecutory delusion with signs consistent with schizophrenia or persistent delusional disorder. The younger sister, Malak, progressively adopted similar delusional beliefs and behaviors within a highly fused sibling relationship, without prior psychiatric history—suggesting a diagnosis of shared psychotic disorder, imposed type. The symptoms persisted despite familial attempts at spiritual healing (roqya). The clinical evolution, relational dynamics, and symptom overlap support the diagnosis of a delusional disorder transmitted from Aya to Malak.

**Conclusions:**

This case highlights the complexity of diagnosing and managing shared psychotic disorder in adolescents. The strong emotional bond, social isolation, and lack of psychiatric insight were key factors in the development of the disorder. Effective treatment requires a multidimensional approach including separation, psychopharmacological intervention, and psychotherapy. Early recognition and targeted intervention are essential to prevent chronic evolution or potential harm.

**Disclosure of Interest:**

None Declared

